# Analysis of the active fraction of Iranian *Naja naja oxiana* snake venom on the metabolite profiles of the malaria parasite by ^1^HNMR *in vitro*

**DOI:** 10.22038/IJBMS.2020.39386.9344

**Published:** 2020-04

**Authors:** Fateme Hajialiani, Taher Elmi, Maryam Mohamadi, Sedigheh Sadeghi, Delavar Shahbazzadeh, Fatemeh Ghaffarifar, Abdolhossein Dalimi, Mohammad Arjmand, Fatemeh Tabatabaie, Zahra Zamani

**Affiliations:** 1 Medical Parasitology Department, School of Medicine -International Campus, Iran University of Medical Sciences, Tehran, Iran; 2 Parasitology and Mycology Department, Faculty of Medicine, Iran University of Medical Sciences, Tehran, Iran; 3 Biochemistry Department, Pasteur Institute of Iran, Pasteur Avenue, Tehran. Iran; 4 Laboratory of Venom and Bio Therapeutics Molecules, Department of Medical Biotechnology, Biotechnology Research Center, Pasteur Institute of Iran, Tehran, Iran; 5 Department of Parasitology, Faculty of Medical Sciences, Tarbiat modares University Tehran, Iran

**Keywords:** Chemometrics, 1HNMR, Metabolomics, Naja naja oxiana venom, Plasmodium falciparum

## Abstract

**Objective(s):**

Malaria is an important parasitic disease with high morbidity and mortality in tropical areas. Resistance to most antimalarial drugs has encouraged the development of new drugs including natural products. Venom is a complex mixture of active pharmaceutical ingredients. The purpose of this study was to investigate the antimalarial activity of purified fractions of *Naja naja oxiana*.

**Materials and Methods:**

Lyophilized venom was purified with a Sephacryl S-200 HR column and the fractions lyophilized and inhibitory concentration 50% against *Plasmodium falciparum* 3D7 *in vitro* obtained. The 4^th^ fraction was run on a Mono Q column, and activity against *P. falciparum* was detected by lactate dehydrogenase assay and purity by SDS PAGE. Large scale culture of the parasite was carried out with and without the active fraction on the ring stage for 48 hr. The parasites were collected and lyophilized and analyzed by 1HNMR. Chemometrics studies were performed using MATLAB, differentiating metabolites were identified by Human Metabolic Database, and metabolic pathways by the Metaboanalyst online package.

**Results:**

The active fraction from the ion exchange column had a 50% inhibitory concentration of 0.026 µg/ml on *P. falciparum in vitro* (*P*<0.001) with molecular weight of 63 kDa by SDS-PAGE and no hemolytic activity. Metabolomics studies on the two groups with and without the fraction identified 5 differentiating metabolites and a number of related pathways.

**Conclusion:**

The metabolites were succinic acid, l-glutamic acid, pyruvic acid, cholesterol, and NAD. The changes in the Krebs cycle and metabolism pathways of nicotinamide and pyruvate were noticeable.

## Introduction

Malaria is one of the indigenous diseases worldwide, especially in tropical countries that has been prevalent since ancient times and has caused substantial damage to human, social, and economic life. WHO reports the death of 445000 people and 216 million cases of malaria (1). Over the past decades, there have been many policies concerning communal health and strategies for controlling and eradicating malaria. Since the year 2000, after enforcing malaria control policies, the number of reported cases has been declining significantly. According to WHO reports, the prevalence of malaria declined by 41% between 2000 and 2015 and the death rate decreased by 62% (2). In a similar manner, from the year 2011 to 2014, the number of malaria cases in Iran decreased from 3572 to 1243 , though it should be noted that 57% of the cases were imported from neighboring countries (3). Resistance to standard anti-malarial drugs still remains a major problem in eradication of malaria (4) and there is a constant search for new natural and synthetic drugs. 

In recent years, with the progress of science, many investigations have been performed on the chemical properties of different types of toxins. Snake venom is a mixture of proteins with different enzymatic properties, consisting of a set of toxic proteins such as neurotoxin, cardio-toxin, cytotoxic, mycotoxin, coagulants, and anticoagulants, enzymes such as proteases, oxidases, phospholipases, and so on. Snake venom as a useful biological source contains several active pharmaceutical compounds that can be used as medications (5).

Castillo *et al.* analyzed the *Bothrops asper snake venom* by ion-exchange chromatography and showed that the phospholipase A2 and its homolog had an anti-plasmodial effect (6). Researchers showed that the *crotamine* polypeptide of South American *rattlesnake* *venom* exhibited cellular penetration properties and was antifungal and antiparasitic. It prevented the development of the *Plasmodium falciparum* parasite in a dose-dependent manner with a 50% inhibitory concentration (IC_50_) of 1.87 μM. *Crotamine* has the ability for selective infiltration into RBCs infected with *P. falciparum* via disturbances in the H+ pool and endangers the metabolism of *Plasmodium*, thus inhibiting parasite growth (7).

Metabolomics simultaneously studies small molecules or the metabolome in a cell, tissue, or an organ and interferes with the primary or secondary metabolism using high throughput technology combined with chemometrics. It also analyses metabolic responses of live systems to pathophysiological stimuli using statistical analysis of biological and Mass spectrometry (MS) and ^1^HNuclear magnetic resonance (^1^HNMR) spectroscopic data. The metabolomics method that was developed by Jeremy Nicholson is the most important direct and simultaneous observation of the physiological state of the cell (8).

A study used ^1^HNMR spectroscopy to ascertain the effectiveness of aqueous extract of cinnamon on *P. falciparum* metabolome *in vitro*. The 50% inhibitory concentration was detected and after metabolite extraction, ^1^HNMR spectroscopy was performed using the NOESY procedure. The data were analyzed by chemometric methods, metabolites were recognized using the Human Metabolome Databases, and the pathways were detected by the Metaboanalyst website. They concluded that the aqueous extract of cinnamon had an IC_50_ value of 1.25 mg/ml on *P. falciparum* *in vitro,* and the differentiating metabolites were succinate acid, glutathione, L-aspartic acid, beta-alanine, glycine, and 2-methylbutyryl. It also affected the main metabolic cycles of alanine, aspartate, glutamate, and pantothenate and biosynthesis of coenzyme A, lysine and glutathione metabolism (9). The most important advantage of metabolomics is the simultaneous analysis of the metabolites and the relationship between them (10).

There seems to be a lack of comprehensive research on the effects of Iranian Cobra snake venom on the pathogenesis of *Plasmodium*. Identification of an active fraction of the venom as a candidate for natural antimalarial drugs would help in the prevention of drug resistance by reducing the use of classical antimalarial drugs. In this report, the effect of the active fraction of Iranian *Naja naja oxiana* snake venom on the metabolite profile of the malaria parasite was studied *in vitro* by ^1^HNMR spectroscopy.

## Materials and Methods

***Venom***

The lyophilized *Naja naja oxiana *toxin was obtained from the Venom and Pharmaceutical Products Laboratory of the Pasteur Institute of Iran.

***Gel chromatography***

Gel filtration chromatography (Sephacryl S-200 HR-HI Prep 16/60, GE America) was used to purify the fractions of the venom. Crude Iranian cobra venom (30 mg) was dissolved in 1 ml of 26 mM ammonium acetate (pH 8.0) (MERCK) and centrifuged for 5 min at 1200 g to remove mucopolysaccharides and particles. After measuring the protein in the supernatant, which was 10 mg/ml, it was injected into the FPLC (GE, Model AKTA) with the S-200 Sephacryl column at a flow rate of 8 ml/min. Fractions were collected manually and the absorption was measured at 280 nm/215 nm and the peaks determined. The run was repeated 6 times and the fractions under each peak from each run were pooled together and lyophilized to concentrate the fractions (Christ Co., Germany), which were then used to determine anti*-*plasmodial activity *in vitro* by microscope counting. 

***Ion exchange chromatography***

The fraction that exhibited the highest antiparasitic effect was purified by ion-exchange chromatography and 36 mg of it was dissolved in 2 ml 20 mM Tris-HCl and NaCl pH 7.5 and 1 ml injected into the Mono Q column (96 x 9.8 cm) of HPLC with a flow rate of 9 ml/min and eluted with gradient of 0 to 9 M NaCl for 86 min and absorption recorded at 280/215 nm. The run was repeated once more and the fractions were then concentrated to 5 ml by dialysis using Amicon Ultra-30 Centrifugal Filter Units and used to determine the anti-plasmodial activity *in vitro *by microscope counting and *Pf*LDH assay. 

***Protein determination***

Protein content was measured from fractions obtained from FPLC chromatography by dissolving the lyophilized proteins in 1 ml DW and HPLC fractions after dialysis with Amicon Ultra-30 Centrifugal Filter Units by the Bicinchoninic Acid method (BCA) using a kit (iNtRON Biotechnology Co. South Korea) according to the manufacturer’s instructions.

***SDS-polyacrylamide gel electrophoresis SDS-PAGE***

Was performed on approximately 15 µl of protein fractions according to the Laemmli method (11) under reducing conditions with 2ME using 12 % resolving gel in Tris-HCl pH 8.8 and 4% stacking gel (pH 6.8) in Tris-glycine buffer pH 8.3 at 95 volts for 3 hr. The gel was stained with Coomassie Blue.

***Hemolytic test***

5 ml of human blood was obtained using heparin as an anticoagulant. Whole blood was washed three times with PBS (pH 7.4) and a suspension of 2% was prepared in normal saline. Serial dilutions of all fractions up to 100 µg/ml from FPLC were prepared in a microplate and 2% RBC suspension (100 μl) added to each well and incubated for 2 hr at 37 ^°^C. The microplate was centrifuged for 10 min at 1200 g (Sigma 3-18k), and the absorbance of the supernatant was determined at 540 nm(12) using normal saline as negative control and triton X100 was used as the positive control. The percent and hemolysis were calculated using this formula:

Percent hemolysis %:100× (OD _test_– OD _negative_ _control_)/ (OD _positive_ _control_ – OD _negative_ _control_)

***MTT assay***

Fibroblast cells HEK293 were first cultured in a 75 ml flask. The cells were then removed from the flask with trypsin, and after counting, 20,000 cells were transferred to each microplate well, after ensuring that the cells were attached to the microplate floor, different concentrations of fractions from 6 μg to 180 ng of each fraction was added to them. After 24 hr, the supernatant of each well was removed, and 100 μl of RPMI 1640 (sans phenol red) and 50 μl of MTT solution were added and kept at 37 ^°^C under 5% CO_2_ for 4 hr. Then, 50 μl of isopropanol solution was added and the plate was mixed in the dark for 15 min, and the absorbance of the samples was recorded at 540 nm (by BioTek Epoch device).***Culture of parasites***

*P. falciparum* parasite, 3D7 strain was cultured according to the procedure of Trager and Jensen (13) in the Biochemistry Department of Pasteur Institute of Iran. Briefly, the parasites were cultured in a medium containing RPMI 1640 medium (Atocel) supplemented with Hepes 5.958 g/l, NaHCO_3_ 2 g/l, gentamicin 25 mg/l, hypoxanthine 13.6 mg/l, and D-glucose 4.5 g/l, along with 10% human serum and 2% hematocrit. The medium was replaced every 48 hr, and the flasks were incubated at 35.5 ^°^C with 3% CO_2_, 5% O_2_, and 90% N_2 (14)_. The media were replaced daily and the growth of the parasite was examined by Giemsa staining of thin smear to verify the stage and percentage of parasitemia by microscopy.

***Synchronization of parasites***

Was carried out at the ring stage after obtaining the pellet by centrifuging the culture at 600 g and adding 4 ml of 5% sorbitol followed by incubation for 10 min at RT with periodic shaking. The pellet was washed 3 times with culture medium and diluted to 5% hematocrit. This procedure was once more carried out after 48 hr. 

***IC***_50_ ***Determination***

When the synchronized parasite number reached 5% in the ring stage, it was diluted to reduce the parasitemia to 0.5% and the hematocrit was adjusted to 1.5%.  This suspension (100 μl) was added to microtiter plate wells in triplicate that contained 90 μl of different concentrations of the fractions of *Naja naja oxiana* venom from FPLC and HPLC with chloroquine as positive control and complete medium as the negative control. The plates were incubated at 37 ^°^C for 48 hr in a CO_2_ incubator. After that time, a thin smear was prepared from each well, stained with Giemsa to determine the percentage of parasitemia and IC_50_ by a microscope using the following formula:

***Growth inhibition percentage***

(parasitemia in control-parasitemia in test)/parasitemia in control X 100 (15).

***Lactate dehydrogenase (LDH) assay***

For estimation of parasitemia, the IC_50_ value was confirmed by detecting the lactate dehydrogenase enzyme activity of *P. falciparum* (16). Briefly according to the procedure of D’Alessandro**, **to each well of a 96-well plate, 100 µl of Malstate solution was added in duplicate and 20 μl of a suspension of medium containing 0.5% parasitemia and 1.5% hematocrit was added together with different concentrations of the active fraction of *Naja naja oxiana* snake venom using chloroquine as positive control and complete medium as negative control. Finally, 25 μl solution of phenazine autosulfate and *nitroblue tetrazolium chloride* was added, and the absorbance was then read at 620 nm.

The results were expressed as the percentage of the viable parasite in drug and control samples by the following formula:

100×(OD_treated sample_ – OD_negative_ _control_)/(OD_positive_ _control_ - OD_negative_ _control_)

***Statistical calculation***

 Was carried out in the above tests using Student’s *t-*test. 

***Preparation of parasites and purification of ring-stage of parasite from RBC on a large scale***

Parasite cultures were grown on a large scale in two groups, with and without active fractions and centrifuged at 1500 rpm for 5 min, supernatant was removed and 20% Saponin was added (40 times the parasite volume) and left for 30 min on ice and then the solution was centrifuged at 4000 rpm for 4 min at 4 ^°^C. The pellet was washed 3 times with PBS (as mentioned above), and the purified parasite pellet was centrifuged in an Eppendorf tube at 12,000 rpm for 5 to 6 min at 4 ^°^C. After removal of the supernatant, the samples were kept at -20 ^°^C.

***Extraction of P. falciparum metabolites***

To the frozen purified parasite, 3 ml saline was added, sonicated and centrifuged for 10 min at 10,000 rpm. The supernatant was removed and for each of 10^8^ cells, 200 λ cold 1.8 mM perchloric acid was added and kept for 1 hr on ice so as to precipitate the metabolites with perchloric acid; the sample was kept on ice for 1 hr and centrifuged at 10,000 rpm for 10 min and the supernatant collected. The pH of the sample was adjusted to 6.8 with KOH(5.4M) and lyophilized. For ^1^HNMR, 1 ml of D_2_O was added to the lyophilized powder and spectroscopy was carried out using NOESY protocol at Central laboratory of technical Isfahan University in a 400 MHz Bruker device for 5 hr (17). 

***Analysis of*** ^1^ ***HNMR spectra***

First, the raw spectra obtained from the ^1^HNMR Bruker were observed using the Mestrec software package and then converted into Excel form for multivariate chemometrics analysis using the Prometab program (version 3_3) on MATLAB (v.7.8.0.347) software. Binning was carried out with 0.004 units on chemical shifts of 0 to 10 ppm and normalized using row-wise normalization, sample median and Pareto Scaling. Principle component analysis was carried out and then PLS-DA, to obtain VIP scores, was performed using the statistical option of the Metabo-analyst website (Metaboanalyst.ca) and chemical shifts of differentiating metabolites were detected and metabolites identified by the Human Metabolome Database at HMDB.Ca. The names of the metabolites were entered into the pathway analysis of the pathway analysis option of Metaboanalyst so as to identify the differentiating metabolic pathways between the two groups.

## Results

**Figure 1:**
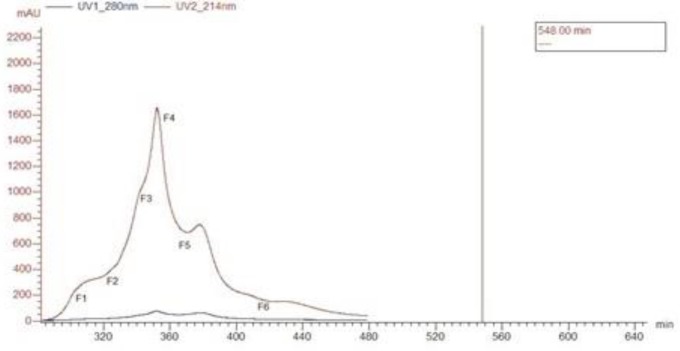
FPC purification of crude venom

The crude venom was purified by FPLC, and its graph is depicted in (Figure 1).

FPLC results of crude venom of *Naja naja oxiana* snake with Sephacryl S-200 column. Fractions obtained from 6 runs were pooled together, and protein estimated in Table 1.

Protein concentrations of the fractions are shown in Table 1.

**Figure 2:**
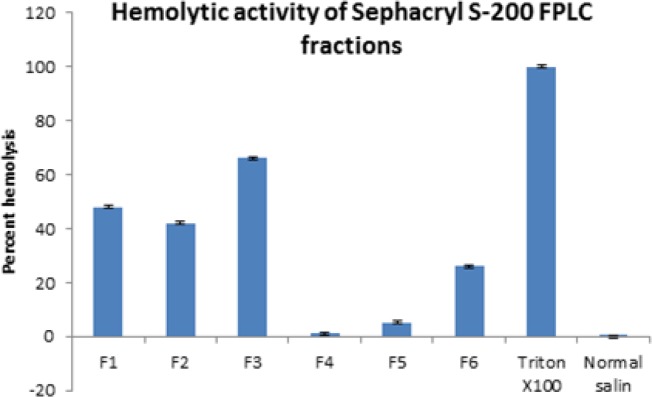
MTT assay of venom fractions from Sephacryl S-200 column on fibroblast HEK 293 cell line

**Figure 3.**
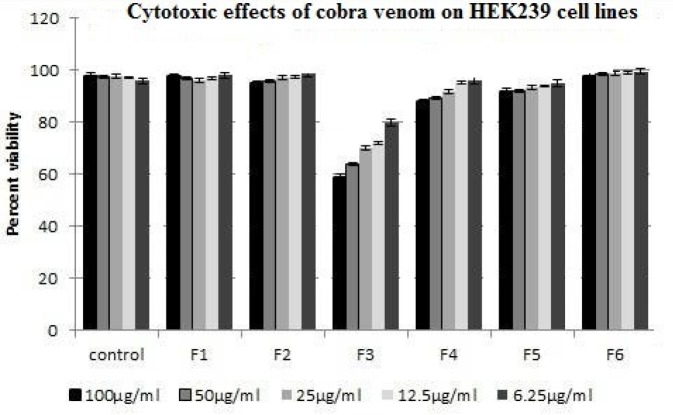
Percent hemolytic activity of venom fractions from SephacrylS-200 at 100 μg/ml

**Figure 4:**
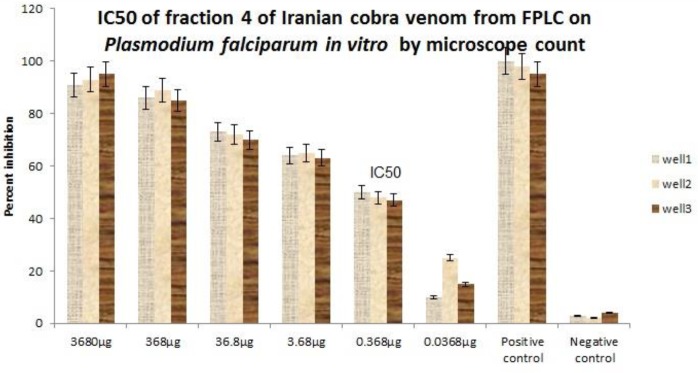
The 4^th^ venom fraction from Sephacryl S-200 had an IC_50_ value of 0.368 µg/ml after 48 hr with significance of *P*<0.001 on *P. falciparum* (ring stage) *in vitro*

 MTT assay showed no toxicity of any fraction at any dilution on HEK293 cells (Figure 2). The hemolytic effect of the fractions at 100 µg/ml is shown in (Figure 3) and the 4^th^ fraction lacked it. The 4^th^ and 5^th^ fractions were tested for anti-plasmodial activity and only fraction 4 exhibited it and its IC_50_ was seen to be 0.368 µg/ml after 48 hr with different dilutions 0.368 µg/ml after 48 hr with the significance of *P*<0.001 (Figure 4).

The 4^th^ venom fraction from Sephacryl S-200 had an IC_50_ value of 0.368 µg/ml after 48 hr with significance of *P*<0.001 on *P. falciparum* (ring stage) *in vitro.*

**Figure 5:**
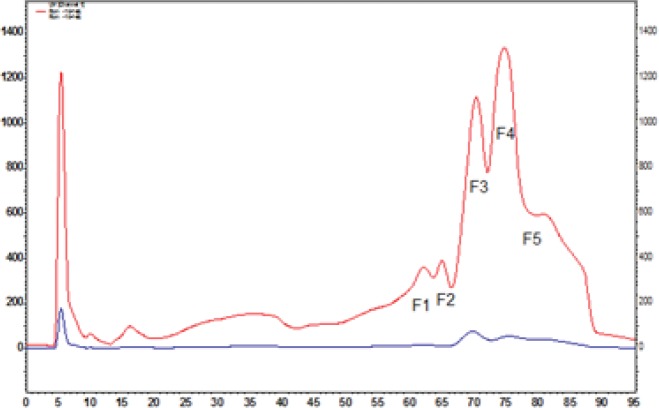
HPLC of F4 fraction of crude *Naja naja oxiana* venom snake with ion-exchange Mono Q column (96 x 9.8 cm). The run was repeated twice and the fractions were pooled

**Figure 6:**
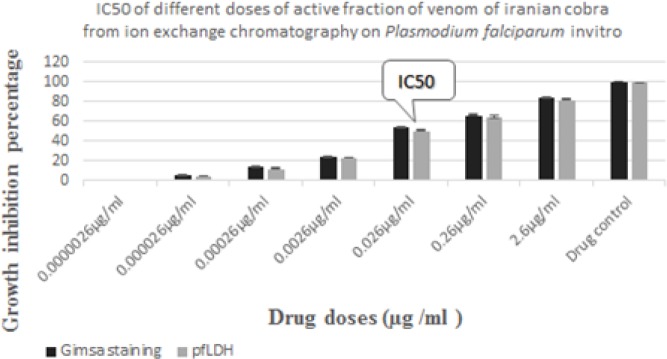
IC_50_ of active fraction from Mono Q column on *P. falciparum in vitro*

The 4^th^ fraction was separated with ion-exchange chromatography (Figure 5) and its protein assayed (Table1) and tested on *P. falciparum in vitro. *The 4^th^ fraction alone had anti-plasmodial activity and its IC_50_ was obtained both by microscopy counting and *Pf*LDH assay (Figure 6), which was 0.026 µg/ml after 48 hr, with the significance of *P*<0.001. This fraction will henceforth be named as “active fraction.” 

**Figure 7:**
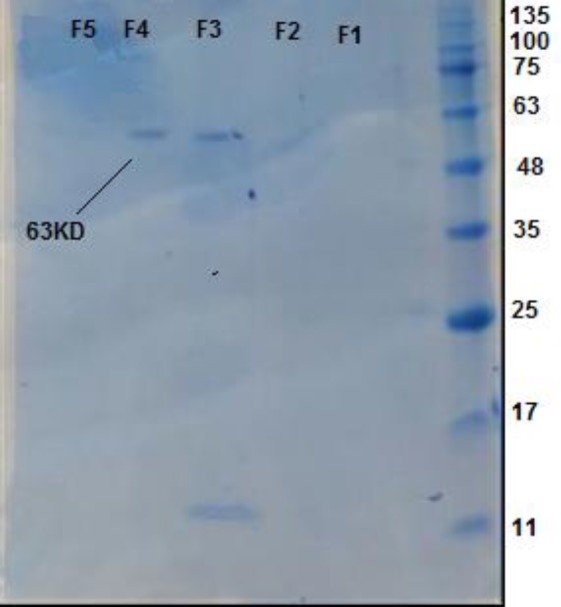
Coomassie blue staining of SDS-PAGE of fractions from ion exchange HPLC with MW markers shown in right hand lane. Lane 4 showed a single band at 63 KD

SDS PAGE of the active fraction with Coomassie Blue depicted a single band at approximately 63 kDa (Figure 7). 

***Metabolomics of P. falciparum parasite in the presence of an active fraction of Naja naja oxiana snake venom in vitro***

**Figure 8:**
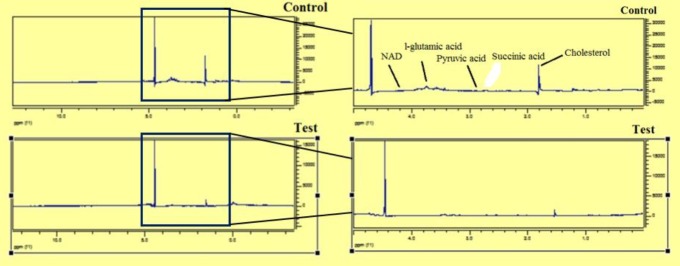
Metabolic spectra of the ring phase in the two groups of control and *Plasmodium falciparum* treated with IC_50_ dose of the active fraction of *Naja naja oxiana* snake venom in vitro. Differentiating metabolites marked inset

^1^HNMR spectra of the peaks of control and active fraction on the ring phase of *P. falciparum* parasite is seen in (Figure 8). 

**Figure 9:**
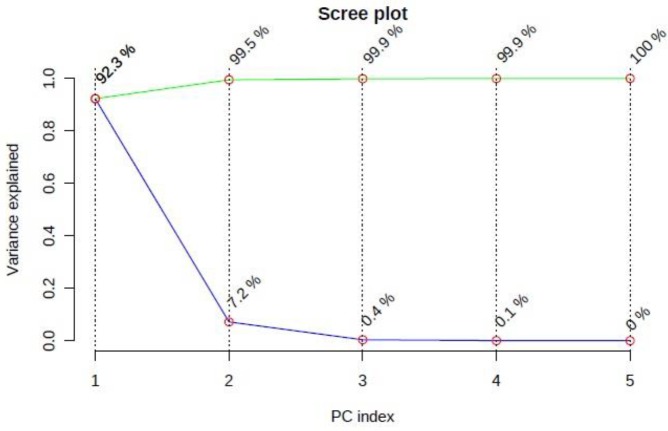
The scree plot shows the variance explained by PCs. The green line on top shows the accumulated explained variance; the blue line underneath shows the variance explained by individual PC

The scree plot from PCA is shown in (Figure 9) with 92% separation in the first component (Figure 9). Score and loading plot of PCA are shown in (Supplement 1, 2).

**Figure 10:**
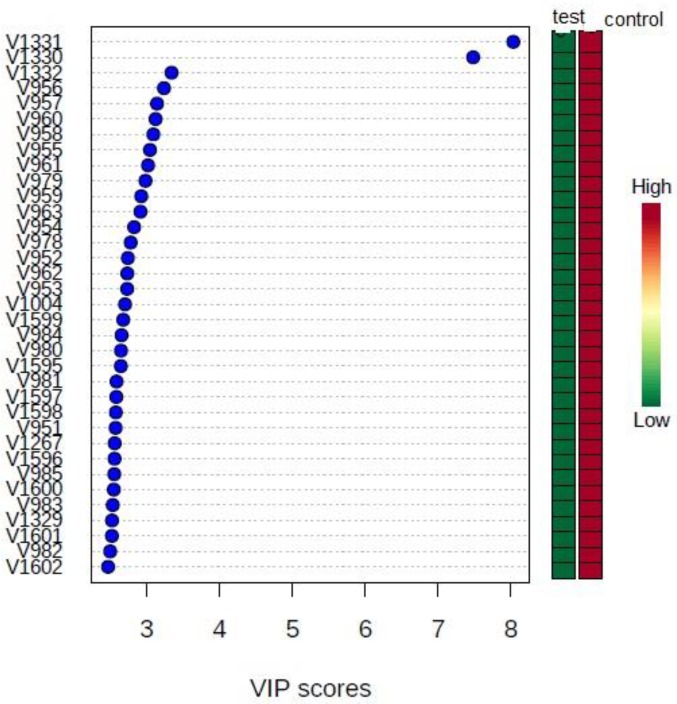
Important features’ VIP scores identified by PLS-DA. Showing the relative amount of differentiating variable metabolites in the two groups of control and *Plasmodium falciparum * treated with IC_50_ dose of active fraction of *Naja naja oxiana *snake venom* in vitro*. The colored boxes on the right indicate the relative concentrations of the corresponding metabolite in each group under study

The variable numbers on the y axis were identified as chemical shifts of metabolites (Figure 10). The first component of PLS-DA showed a separation of 92.1% with their R^2^and Q^2^ values (Table 2).

Metabolites with their chemical shifts were identified from HMDB (Table 3). 

**Figure 11:**
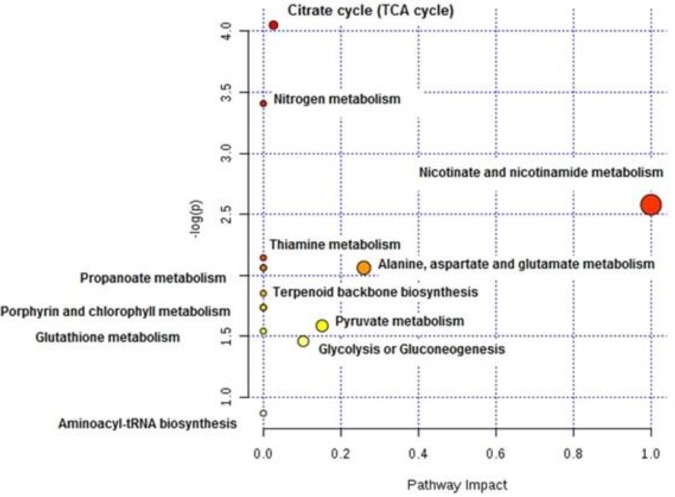
Graph showing the metabolic pathway impact of differentiating pathways in control and *Plasmodium falciparum* treated with IC_50_ dose of active fraction of *Naja naja* groups. The x-axis is the pathway impact and the y-axis –log P. Pathway impact between the two groups measured by node importance according to relative betweenness centrality; circles larger, higher, or closer to the axis are of higher significance. The impact is the pathway impact value calculated from the pathway topology analysis. Calculations were carried out using the Metaboanalyst website

The main metabolomic pathways identified by Metaboanalyst website are seen in Table 4 with their *P-*values in pathway analysis and depicted in Figure 11.

## Discussion

Venom is considered a biological source of several active pharmaceutical compounds. They include enzymes such as phospholipases, proteases, oxidases, other proteins like disintegrin, neurotoxins, *etc.,* and peptides and it is this complex composition that is responsible for its deadly effects on the vital systems of the body. The highest content of enzymes is observed in the snake venoms of which there is only a single oxidase, L-amino acid oxidase, but hydrolases are in abundance which includes phospholipases А2, proteinases/peptidases, acetylcholinesterase, and hyaluronidases. Non-enzymatic proteins are more varied and it is seen that in snakes, it includes the three-finger toxins, proteinase inhibitors, disintegrins, nerve growth factor, *etc. *(18). 

Sherman found that the drug ancrod, derived from the Malaysian rattlesnake, has a blood-thinning effect and can be used to treat patients immediately after cerebral strokes (19). Snake venom is also reported to have antitumor activity (20) and has potential for treatment of cancer as tested on stomach, colon, adenocarcinoma, and leukemia cell lines among others (21) . The venom of the Chinese snake *Naja naja atra* was found to selectively act on sarcoma cells and confirmed to have antitumor activity, especially on malignant tumors of the gastrointestinal tract and gastric cancer (22).

L-amino oxidase is the only oxidase enzyme present in the snake venom with important applications. L-amino-oxidase isolated from pit viper venom *Bothrops jararaca *is the precursor f*or *Captopril, which is an angiotensin-converting enzyme inhibitor approved by the USA Food and Drug Administration in 1981 (23).

L-amino-oxidase purified by ion-exchange chromatography and hydrophobic chromatography from *Naja naja oxiana *is also shown to have antibacterial properties and inhibits the growth of gram-positive bacteria *Bacillus subtilis* and gram-negative bacteria *Escherichia coli* (24).

Researchers discovered that a phospholipase A2 (PLA2) from the eastern diamondback rattlesnake (*Crotalus adamanteus*) inhibits ookinete adhesion and oocyst formation. They have also shown that the *Crotalus adamanteus *PLA2 greatly reduces the efficiency of the oocyst formation of malaria parasites (25).

Crotamin is a small peptide isolated from the venom of the South American rattlesnake *Crotalus durissus terrificus* and is reported to have anti-plasmodial and antileishmanial activity. Its mechanism is suggested to be the potential involvement of disruption of H+ homeostasis in *P. falciparum* parasites (6).

There has been no report of the anti-plasmodial activity of the Iranian cobra venom as yet. Hence, this investigation was carried out by purification of the venom of the Iranian *Naja naja oxiana* and its effect on the ring stage of *P. falciparum.* The venom fraction had no toxic effect on live cells* in vitro* as seen in MTT studies on fibroblast cells. Also, the active fraction did not have any hemolytic activity. In this study, the metabolic profile of the *P. falciparum* was also analyzed by ^1^HNMR spectroscopy. The venom of *Naja naja oxiana* was purified by two chromatographies and the active fraction did not have any hemolytic effect. It is interesting that the two methods, both Giemsa staining and LDH assay, showed similar results. The active fraction had an IC_50_ value of 0.026 µg/ml much higher than the 0.078 µg/ml reported for crotamine from South American rattlesnake venom on *P. falciparum,* which seemed to make it more potent (7). Crotamine is a small peptide of 4.8 kDa and the dose for IC_50 _would be higher as compared to the active fraction having 63 kDa molecular weight. PLA2 is reported to have strong inhibitory effects on the sexual stage of the parasites (25), but its presence is unlikely in the active fraction from Iranian cobra as this is the main enzyme responsible for hemolysis and has a molecular weight of 14 kDa. The active fraction obtained from Iranian cobra venom did not have any hemolytic activity and demonstrated a single line of high molecular weight as shown by Coomassie Blue staining and only 0.9% hemolytic activity and so it is improbable to contain the phospholipase A2 enzyme (26).

This is the first metabolomics study on the activity of snake venom on *P. falciparum* parasites to date. Metabolomic changes in *P. falciparum* in the control and active fraction treated group were detected by ^1^HNMR spectroscopy using chemometric methods. The metabolites identified participated in a number of pathways (Table 4), the most important of which will be discussed.

 The first cycle is the Krebs cycle in which succinic and pyruvic acids participate. This pathway is closely linked to the pyruvate metabolism in which the same metabolites are present. Although, early studies have shown that the parasite’s blood stage is almost entirely dependent on glucose fermentation for energy production and minimal oxygen consumption, and the parasite can also convert glucose into lactate by the glycolysis process during metabolic phases, the parasite genes code all the enzymes needed in the Krebs cycle, which means it has all the enzymes needed for the TCA cycle (27). A study in 2010 showed that the metabolism of the Krebs cycle in *P. falciparum* is largely separate from glycolysis. The main source of carbon for the Krebs cycle is aspartate, asparagine, glutamate, and glutamine amino acids that are produced by the breakdown of the hemoglobin molecule that can be de-aminated and converted to oxaloacetate or alpha-ketoglutarate. They believed that the Krebs cycle in this parasite is not like a cycle but has a branched structure, and these branches in several reactions act in the opposite direction of the standard pathway of the Krebs cycle (28), however this theory has been refuted (29). The flow of carbon in the mitochondria of the parasite is divided into two independent branches. One of the branches that run counterclockwise begins with carboxylation and reduction of oxoglutarate to isocitrate. Isocitrate then is converted to citrate and produces two carbons Acetyl Co A and oxalate acetate and finally, oxaloacetate converts to malate (28). The second branch in the Krebs cycle acts in clockwise direction and, during several reactions, can oxidize oxoglutarate to succinyl-CoA, succinyl-CoA to succinate (succinic acid), succinic acid to fumarate, and ultimately fumarate to malate. The Acetyl-CoA with two carbons is the product of this branch that is used for histone acetylation. 

Also, acetyl CoA can be obtained from *phosphoenolpyruvate*, which is a product of glycolysis in the apicoplast of the parasite, and is used for acetylation of amino sugars (28). Glucose consumption in red blood cells infected with *P. falciparum* parasites is more than in uninfected red blood cells. Non-sexual proliferation of parasites is also faster. It should be noted that reproductive energy in the non-sexual phase is provided by the TCA cycle, and in sexual phase is provided through glucose because proliferation of the gametocytes, in contrast to non-sexual proliferation, is sensitive to aconitase inhibitor.  This could be a reason for developing drug resistance. During the glycolysis pathway (one of the pathways detected), not only energy but also pyruvate is produced. During the TCA pathway, energy is also generated; that is malaria parasite obtains energy from these two pathways. This cycle is indispensable for intracellular growth of the parasite(30). The active fraction from the Iranian cobra seems to act on this cycle. There have also been reports of a study of the action of *Crotalus durissus terrificus *venom on rat macrophage metabolism and function and it was seen that it increased activities of key enzymes of glycolysis and glutaminolysis besides H_2_O_2_ and NO production and candidacidal activity (31). 

Nicotine amide metabolism pathway is the next important one detected. Nicotinamide dinucleotide (NAD^+^) is a necessary metabolite for the malaria parasite, which is used as a reducing cofactor and enzyme-substrate in multiple cellular processes. NAD^+^ levels have been observed in the red blood cell infected with *Plasmodium falciparum*, but little is known about how the parasite produces NAD. In this study, it was found that the active fraction of *Naja naja oxiana* can effect this metabolite, and since the metabolic role of NAD^+^ is very important for the parasite, it can be a good drug target for antimalarial compounds (32).

The next significant pathway altered is the metabolism of alanine, aspartate, and glutamate in the *P. falciparum* parasite. Our data indicate that significant changes occurred in the metabolic pathways of alanine, aspartate, and glutamate specifically in the *adenyl-succinic* acid pathway. Previous studies indicated that the level of glutamine that is a by-product of this metabolic pathway increased in the brains of mice infected with malaria while glutamine level decreased in the serum (33). Also, oral administration of venom of *Naja* *naja* altered the aminoacid enzyme levels, such as aspartate aminotransferase, alanine aminotransferase, and glutamate dehydrogenase in liver cells of mice (34). 

Pyruvic acid participates in terpenoid backbone biosynthesis, the next important pathway. Terpenoids are also referred to as isoprenoids, which are hydrocarbons that participate in vital cellular processes. There are reports of *P. falciparum* terpenoids being used in t-RNA isoprenylation and protein including vitamin E synthesis, carotenoids, ubiquinone, and dolichols. It is interesting that in the plasmodium isoprenoid synthesis via the methylerythritol phosphate (MEP) pathway is an appealing target for the development of new antimalarials. A new regulator of the MEP pathway in *P. falciparum* is detected as HAD1 (PfHAD1) (PlasmoDB ID PF3D7_1033400), a sugar phosphatase member of the haloacid dehalogenase-like hydrolase (HAD) superfamily, which is detected as a negative regulator that acts upstream from the MEP pathway and could be further studied as a likely drug target against malaria (35).

Glutathione metabolism is an important one altered by *Naja naja oxiana* venom. *P. falciparum*, as a trophozoite in the human red blood cells, shows a severe glutathione metabolism. The amino acid glutamine has been shown to participate in a number of cycles, especially glutathione, which not only plays an important role in the antioxidative defense but helps in keeping the cytosolic medium at a low pH. Many known glutathione-associated processes are based on parasite-specific lifestyle. Glutathione reduction enables the speed of cell growth by providing electrons for deoxyribonucleotide synthesis and in sections of heme detoxification, that is, supports the product of hemoglobin digestion. Generally, free radicals in the parasite can eliminate the sequential reactions involved in GS radicals and GS thiolates (36).

Glutathione is conjugated to the indigestible compounds such as antimalarial drugs, as glutathione S-transferase substrate, it is a well-known detoxification cycle. It is the coenzyme of the glyoxalase system that neutralizes methylglyoxal responsible for increasing the glycolysis in the trophozoite. Other proteins involved in GSH-dependent processes include glutathione reductase, glutaredoxin, glyoxalase 1 and 2, glutathione-s-transferase, and thioredoxin. These proteins and ATP-dependent enzymes of glutathione synthesis were studied as factors in the pathophysiology of malaria and drug targets. Methylene Blue is a well-known structural inhibitor of glutathione reductase of *P. falciparum* and its combination with chloroquine is expected to be a promising drug for malaria (37). It is interesting that rat liver cells administered *Naja haje* venom showed reduction of glutathione, catalase, glutathione reductase, and glutathione-S-transferase activities (38). 

Biosynthesis of the aminoacyl tRNA synthase in the *Plasmodium-falciparum* parasite is the next pathway altered by the venom tested. This enzyme is made in two steps by the amino-acyl synthase. There are at least 20 types of these enzymes in each cell. These enzymes play an important role in increasing the accuracy of protein synthesis. In eukaryotes and prokaryotes, methionine and formyl-methionine are the primary amino acids in the protein synthesis, respectively. If the activation of amino acids is mistaken or there is a problem in the amino acid structure, in the place called editing site, the link between t-RNA and amino acid hydrolyzes, thereby preventing the amino acid from entering into the protein structure.

The nucleus of the *P. falciparum* has two codes for methionyl t-RNA synthetase that are called *pfmrscy* and *pfmrsapi *and *pfmrsapi* in the apicoplast of the malaria parasite. This enzyme has been considered for many years a drug target for bacteria and fungi, and in recent years, considerable research has been done on the objective of this enzyme in eukaryotes as a drug target (39). It is hoped that in the coming years, this enzyme is available as a drug target in parasites. Pham *et al.,* over the past decade, have investigated the amino-acyl tRNA synthetase in eukaryotic parasites. The soluble crystalline structure of parasitic t-RNA synthase is a good drug target for parasites such as *P. falciparum* (40).

In this report, we showed that in the *P. falciparum* parasite, the active fraction of snake venom could cause changes in this enzyme. Our data showed that changes have likely occurred in the metabolites of l-isoleucine, l-leucine, l-histidine, and l-cysteine in the metabolism of aminoacyl-tRNA biosynthesis. 

**Table 1.** Protein concentration of fractions from columns by the BCA method

Fractions from ion-exchange HPLC		Fractions from FPLC	
Protein concentration in mg/ml	Name	Protein concentration in mg/ml	Name
0.03	F1	0.69	F1
0.03	F2	3.4	F2
0.5	F3	8.3	F3
2.6	F4	36.86	F4
0.03	F5	5.75	F5
		0.8	F6

**Table 2:** PLS-DA classification using different numbers of components

Q^2^	R^2^	Accuracy	Explained variance	
-1	0.2	0.3	92.1%	Component 1
-2	0.25	0.2	7.2%	Component 2
-2	0.5	0.7	0.6%	Component 3

**Table 3:** Metabolites identified from their chemical shifts

Numbers	Variation No.	Metabolite Name	Chemical shift	HMDB No.	Flux	Score values
1	V-979	NAD	4.225	HMDB0000067		2.0
2	V-1186 V-1187	Pyruvic acid	2.534	HMDB0000243		2.0
3	V-956 V-957 V-958	Glutamic acid	3.736	**HMDB0000148**		3.0
4	V-1267	Succinic acid	2.393	**HMDB0000254**		2.0
5	V-1330 V-1331 V-1332	Cholesterol	1.854	**HMDB0000067**		7.5

**Table 4:** The table depicts the differentiating metabolites and pathways in the control and treated *Plasmodium falciparum* with IC_50_ dose of active fraction of *Naja naja oxiana*

Numbers	Metabolic pathways	Total	Expected	Hits	Raw *P*
1	Citrate cycle (TCA cycle)	20	0.22272	2	0.017413
2	Nitrogen metabolism	3	0.033408	1	0.03311
3	Nicotinate and nicotinamide	7	0.077951	1	0.075886
4	Propanoate metabolism	12	0.13363	1	0.12721
5	Alanine, aspartate and glutamate metabolism	12	0.13363	1	0.12721
6	Pyruvate metabolism	20	0.22272	1	0.20457
7	Glycolysis or gluconeogenesis	23	0.25612	1	0.23213
8	Thiamine metabolism	11	0.12249	1	0.11714
9	Terpenoid backbone biosynthesis	15	0.16704	1	0.1569
10	Porphyrin and chlorophyll metabolism	17	0.18931	1	0.17623
11	Glutathione metabolism	21	0.23385	1	0.21384
12	Aminoacyl-tRNA biosynthesis	46	0.51225	1	0.41899

The second column shows the 5 metabolites that participate in the differentiating pathways, with their significance as detected by the Metaboanalyst website. It can be noted that metabolites can be detected in more than one pathway. Since different pathways were tested at the same time, the statistical *P*-values from enrichment analysis were further adjusted for multiple testing. In particular, the total is the total number of compounds participating in the pathway; the hits are the actually matched number from the user uploaded data; the raw *P* is the original *P*-value calculated from the enrichment analysis

## Conclusion

It can be concluded that the active fraction of *Naja naja oxiana* venom has an inhibitory effect on *P. falciparum* *in vitro *with IC_50_ of 0.026 µg/ml with significance of *P*<0.001. This is the most potent anti-plasmodial snake venom fraction reported so far. The altered metabolites are succinic acid, l-glutamic acid, pyruvic acid, cholesterol, and NAD as per the metabolomics study. The data in this report demonstrated that in the ring phase, the active venom fraction mostly affects the metabolism of amino acids and the pathways related to the biosynthesis of proteins and isoprenoids. It is for the above reasons that the changes in the Krebs cycle and metabolism pathways of nicotinamide and pyruvate are noticeable. 

## References

[1] Organization WHO (2017). World malaria report.

[2] Organization WHO (2015). World malaria report.

[3] Norouzinejad F, Ghaffari F, Raeisi A (2016). Epidemiological status of malaria in Iran, 2011–2014. Asian Pac J Trop Med.

[4] Elmi T, Hajialiani F, Asadi M R, Orujzadeh F, Kalantari Hesari A, Rahimi Esboei B et al (2019). A Study on the Effect of Zingiber Officinale Hydroalcoholic Extract on Plasmodium berghei in Infected Mice: An Experimental Study. JRUMS.

[5] Thangam R, Gunasekaran P, Kaveri K, Sridevi G, Seundarraj S, Paulpandi M (2012). A novel disintegrin protein from Naja naja venom induces cytotoxicity and apoptosis in human cancer cell lines in vitro. Process Biochem.

[6] Castillo J, Vargas L, Segura C, Gutiérrez M, Pérez JC (2012). In vitro antiplasmodial activity of phospholipases A2 and a phospholipase homologue isolated from the venom of the snake Bothrops asper. Toxins.

[7] Maluf S, Dal Mas C, Oliveira E, Melo P, Carmona A, Gazarini M (2016). Inhibition of malaria parasite Plasmodium falciparum development by crotamine, a cell penetrating peptide from the snake venom. Peptides.

[8] Zerez C, Roth E, Schulman S, Tanaka K (1990). Increased nicotinamide adenine dinucleotide content and synthesis in Plasmodium falciparum-infected human erythrocytes. Blood.

[9] Parvazi S, Sadeghi S, Azadi M, Mohammadi M, Arjmand M, Vahabi F (2016). The effect of aqueous extract of cinnamon on the metabolome of Plasmodium falciparum using 1HNMR spectroscopy. J Trop Med.

[10] Griffin JL (2003). Metabonomics: NMR spectroscopy and pattern recognition analysis of body fluids and tissues for characterisation of xenobiotic toxicity and disease diagnosis. Curr Opin Chem Biol.

[11] Laemmli UK (1970). Cleavage of structural proteins during the assembly of the head of bacteriophage T4. Nature.

[12] Memar B, Jamili S, Shahbazzadeh D, Bagheri K (2016). The first report on coagulation and phospholipase A2 activities of Persian Gulf lionfish, Pterois russelli, an Iranian venomous fish. Toxicon.

[13] Trager W, Jensen JB (1976). Human malaria parasites in continuous culture. Science.

[14] Radfar A, Méndez D, Moneriz C, Linares M, MarínGarcía P, Puyet A (2009). Synchronous culture of Plasmodium falciparum at high parasitemia levels. Nat Protoc.

[15] Tasanor O, Noedl H, Na-Bangchang K, Congpuong K, Sirichaisinthop J (2002). An in vitro system for assessing the sensitivity of Plasmodium vivax to chloroquine. Acta Trop.

[16] D’alessandro S, Silvestrini F, Dechering K, Corbett Y, Parapini S, Timmerman M (2013). A Plasmodium falciparum screening assay for anti-gametocyte drugs based on parasite lactate dehydrogenase detection. J Antimicrob Chemother.

[17] E.D B ( 2007). High Resolution NMR. 2.

[18] Utkin YN (2015). Animal venom studies: Current benefits and future developments. World J Biol Chem.

[19] Sherman D (2016). Drug Derived From Snake Venom May Help Stroke Patients. Jama Jam Med Assoc.

[20] Liu C, Yang H, Zhang L, Zhang Q, Chen B, Wang Y (2014). Biotoxins for cancer therapy. Asian Pac J Cancer Prev.

[21] Dhananjaya B, Sivashankari PJ (2015). Snake venom derived molecules in tumor angiogenesis and its application in cancer therapy; an overview. Curr Top Med Chem.

[22] S Liberio M, A Joanitti G, Fontes W (2013). Anticancer peptides and proteins: a panoramic view. Protein Pept Lett.

[23] ROY A (2011). Structural and Functional Characterization of Haditoxin, a novel neurotoxin isolated from the venom of Ophiohagus Hannah (King Cobra). J Biol Chem.

[24] Samel M, Tõnismägi K, Rönnholm G, Vija H, Siigur J, Kalkkinen N (2008). L-Amino acid oxidase from Naja naja oxiana venom. Comp Biochem Physiol B Biochem Mol Biol.

[25] Zieler H, Keister D, Dvorak J, Ribeiro JM (2001). A snake venom phospholipase A2 blocks malaria parasite development in the mosquito midgut by inhibiting ookinete association with the midgut surface. J Exp Biol.

[26] Tischfield J (1997). A reassessment of the low molecular weight phospholipase A2 gene family in mammals. J Biol Chem.

[27] Ginsburg HJ (2010). Metabolism: Malaria parasite stands out. Nature.

[28] Olszewski K, Mather M, Morrisey J, Garcia B, Vaidya AB, Rabinowitz J (2010). Branched tricarboxylic acid metabolism in Plasmodium falciparum. Nature.

[29] Olszewski K, Llinás M (2011). Central carbon metabolism of Plasmodium parasites. Mol Biochem Parasitol.

[30] Roth J (1990). Plasmodium falciparum carbohydrate metabolism: a connection between host cell and parasite. Blood Cells.

[31] Sampaio S, SousaeSilva M, Borelli P, Curi R, Cury Y (2001). Crotalus durissus terrificus snake venom regulates macrophage metabolism and function. J Leukoc Biol.

[32] O’Hara J, Kerwin L, Cobbold S, Tai J, Bedell T, Reider P (2014). Targeting NAD+ metabolism in the human malaria parasite Plasmodium falciparum. PLoS One.

[33] Ghosh S, Sengupta A, Sharma S, Sonawat H (2012). Metabolic fingerprints of serum, brain, and liver are distinct for mice with cerebral and noncerebral malaria: a 1H NMR spectroscopy-based metabonomic study. J Proteome Res.

[34] Malleswari M, Josthna P, Doss PJ (2015). Orally administered venom of Naja naja alters protein metabolic profiles in the liver of albino rats. Int J Life Sci Biotechnol Pharma Res.

[35] Guggisberg A, Amthor R, Odom AR (2014). Isoprenoid biosynthesis in Plasmodium falciparum. EC. Eukaryot Cell.

[36] Müller T, Johann L, Jannack B, Brückner M, Lanfranchi DA, Bauer H (2011). Glutathione reductase-catalyzed cascade of redox reactions to bioactivate potent antimalarial 1, 4-naphthoquinones–a new strategy to combat malarial parasites. J Am Chem Soc.

[37] Meissner PE, Mandi G, Witte S, Coulibaly B, Mansmann U, Rengelshausen J (2005). Safety of the methylene blue plus chloroquine combination in the treatment of uncomplicated falciparum malaria in young children of Burkina Faso. Malar J.

[38] Al-Quraishy S, Dkhil M, Moneim A (2014). Hepatotoxicity and oxidative stress induced by Naja haje crude venom. J Venom Anim Toxins Incl Trop Dis.

[39] Hussain T, Yogavel M, Sharma AJ (2015). Inhibition of protein synthesis and malaria parasite development by drug targeting of methionyl-tRNA synthetases. Antimicrob Agents Chemother.

[40] Pham J, Dawson K, Jackson K, Lim E, Pasaje C, Turner K (2014). Aminoacyl-tRNA synthetases as drug targets in eukaryotic parasites. Int J Parasitol Drugs Drug Resist.

